# Characterization of genital chlamydia trachomatis infection among women attending infertility and gynecology clinics in Hunan, China

**DOI:** 10.1186/s12879-024-09254-8

**Published:** 2024-04-15

**Authors:** Qianting Zhou, Jiayan Li, Lipei Luo, Shuling Min, Li Wang, Lixiu Peng, Yinglan Hou, Ping He, Song He, Shixing Tang, Hongliang Chen

**Affiliations:** 1 Hengyang Medical School, The Affiliated Chenzhou Hospital, Chenzhou No. 1 People’s Hospital, University of South China, Chenzhou, China; 2 Department of Epidemiology, School of Public Health, Southern Medical University, Guangzhou, China; 3 Clinical Laboratory, Shenzhen Qianhai Shekou Free Trade Zone Hospital, Shenzhen, China

**Keywords:** *Chlamydia* trachomatis, Genotype, Seroprevalence, Luciferase immunosorbent assays, Cervical intraepithelial neoplasia

## Abstract

**Background:**

Genital infection with *Chlamydia trachomatis* (*C. trachomatis*) is a major public health issue worldwide. It can lead to cervicitis, urethritis, and infertility. This study was conducted to determine the characteristics of genital *C. trachomatis* infection among women attending to the infertility and gynecology clinics.

**Methods:**

Endocervical swabs were collected from 8,221 women for *C. trachomatis* nucleotide screening and genotyping, while serum samples were collected for *C. trachomatis* pgp3 antibody determination using luciferase immunosorbent assays.

**Results:**

High *C. trachomatis* DNA prevalence (3.76%) and seroprevalence (47.46%) rates were found, with genotype E (27.5%) being the most prevalent. *C. trachomatis omp1* sense mutation was associated with cervical intraepithelial neoplasia (CIN) (odds ratio [OR] = 6.033, 95% confidence interval [CI] = 1.219–39.185, *p* = 0.045). No significant differences in *C. trachomatis* seroprevalence rates were observed between women with detectable *C. trachomatis* DNA in the infertility and routine physical examination groups (86.67% vs. 95%, *p* > 0.05); however, among women with negative *C. trachomatis* DNA, the former group had a markedly higher seroprevalence than the latter group (56.74% vs. 20.17%, *p* < 0.001). *C. trachomatis* DNA, but not pgp3 antibody, was significantly associated with CIN (OR = 4.087, 95% CI = 2.284–7.315, *p* < 0.001).

**Conclusion:**

Our results revealed a high prevalence, particularly seroprevalence, of *C. trachomatis* among women with infertility. Furthermore, we found an association between *C. trachomatis omp1* sense mutations and CIN. Therefore, *C. trachomatis* serves as a risk factor for CIN.

**Supplementary Information:**

The online version contains supplementary material available at 10.1186/s12879-024-09254-8.

## Background

*Chlamydia trachomatis* one of the most common sexually transmitted bacterial infections worldwide [1]. According to the World Health Organization, *C. trachomatis* affects approximately 400 million people worldwide. However, the actual number of infected individuals may be higher as *C. trachomatis* infection is often asymptomatic in 50% of men and 80% of women [2]. *C. trachomatis* infection is more prevalent in women than in men, and its prevalence varies with age and country of residence. With a unique biphasic developmental cycle, *C. trachomatis* can cause non-gonococcal urethritis and pelvic inflammatory disease, leading to ectopic pregnancy. As a result, it is gradually becoming an important public health issue for women [2]. In some developed countries, *C. trachomatis* screening programmes have been implemented to reduce its transmission and reproductive tract complications. In Sweden, extensive Chlamydia screening of asymptomatic young women in a variety of health care settings were recommended by Swedish Institute for Infectious Disease Control, and all testing and treatment are free of charge. Moreover, the Communicable Diseases Act has made it mandatory to report *C. trachomatis* genital infections including contact tracing, mandatory partner notification, and compulsory testing of suspicious partners [3]. In 1979, the US Centers for Disease Control established the first training center and model clinics for sexually transmitted disease prevention and recommended annual screening of all sexually active women aged ≤ 25 years [4]. Following large-scale screening of asymptomatic women, the prevalence of *C. trachomatis* has reduced in developed countries to some extent, but not in low-resource settings, such as Africa and Asia, including Burundi, India, and poor regions of China.

*C. trachomatis* genotyping is useful for monitoring re-infections and treatment efficacy. Furthermore, it provides useful information for the clinical treatment and vaccine development for *C. trachomatis* infection [3]. *C. trachomatis* is classified into 19 serovars on the basis of antibody specificity toward the major outer membrane protein (MOMP). These serovars exhibit distinct tissues tropism [5], e.g., serovars A–C cause trachoma, serovars D–K cause oculogenital infections, and serovars L1–3 cause lymphogranuloma venereum. The major outer membrane protein of *C. trachomatis* is encoded by a single copy gene of *omp1*, which differs across serovars [6]. This protein is an immunodominant antigen and contains four variable segments (VS1–4) [2, 7], which are flanked and interspaced with five constant domains [8]. Although several studies have described the *omp1* mutation sites [6, 9], their relationship with the clinical manifestations remains unclear.

The use of antibodies to identify *C. trachomatis* infections often underestimates their prevalence due to the inability to differentiate between recently acquired and previous infections. Furthermore, the likelihood of seropositivity increases with the cumulative number of infections [10]. Pgp3 is highly conserved across isolates and rarely found in *C. pneumonia*, which was recognized as a sensitive and unique serum antibody biomarker of *C. trachomatis* infection due to the capability to avoid cross-reactivity with other *Chlamydia spp* [11]. Besides, 98.7% sequence identity of Pgp3 among different *C. trachomatis* serovars maintains the cross-reactivity within *C. trachomatis* species [12]. Pgp3-based serological testing was then widely used to monitor *C. trachomatis* infections among younger children and predict the development of infertility due to tubal dysfunction [13]. Thus, knowledge regarding the seroepidemiology of *C. trachomatis* is important for molecular epidemiological investigation and determining the prevalence and incidence of infections, for differentiating between recent and past infections, and for identifying subclinical infections.

Our previous studies have shown that *C. trachomatis* infection is associated with low-grade intraepithelial neoplasia [14]. The clinical manifestations of *C. trachomatis* infection vary with the genotype, i.e., almost half of the asymptomatic patients have been infected with serovar E. Infections with serovars F and G are closely associated with a young age and lower abdominal pain, respectively [15]. However, the effects of *C. trachomatis* pgp3 antibody, *omp1* genotype, and *omp1* gene mutation (sense and nonsense mutations) on cervical intraepithelial neoplasia (CIN) and vaginal inflammation have not been well-elucidated.

Our previous studies revealed that women with infertility have a similar *C. trachomatis* prevalence compared to healthy women attending physical examination center (PEC). *C. trachomatis* infection is a significant risk factor for female infertility. Although *C. trachomatis* infections of the female genital tract may recover spontaneously over several days, reinfection occurs in 10–20% of cases, typically within 12 months [16]. Although it is difficult to detect *C. trachomatis* DNA among women with infertility and passive infection, the antibody can persist for a long period. Accordingly, we speculated that women with infertility may have a high *C. trachomatis* seroprevalence, representing both recently acquired and previous infections. To test this, we enrolled a large number of women from the infertility and gynecology clinics and PEC to assess the prevalence of *C. trachomatis* infection, its subtype distribution, *omp1* mutations, seroprevalence, and associated cervical lesions.

## Methods

### Ethics approval and consent to participate

All participants provided written informed consent for participation in this study. For those who were under the age of 16, the informed consents were provided by the parents or legal guardians. This study was performed in accordance with the Declaration of Helsinki, and the study protocol was approved by the Ethics Committee of Chenzhou No. 1 People’s Hospital (CZ/1128).

### Participants and clinical samples

This study included women aged ≥ 14 years who visited the PEC or infertility and gynecological clinic of a teaching hospital in Chenzhou between July 2019 and January 2022. This hospital is the largest general hospital with more than 4,000 beds in Chenzhou and receives patients from the entire city and nearby regions. The inclusion criteria included: (1) non-menstruating women; (2) women with infertility: women of childbearing age who have not become pregnant within 1 year, despite couple cohabitation, normal sexual life, and no contraceptive measures. Seven women with infertility aged 17–19 who were unmarried, but engaged, were also included in the infertility group; (3) women who were not treated with the following drugs within 3 days before the examination: antibiotics, antiviral drugs, and vaginal medications; and (4) women with no sexual activity within 24 h. The exclusion criteria were: (1) refusal to provide informed consent; (2) any acute or chronic condition that would limit the ability of the patient to participate in the study; and (3) women diagnosed with infertility during the annual physical examination in the PEC.

Cervical swab samples were obtained by physicians as described previously [14, 17]. *C. trachomatis* DNA was extracted and purified within 48 h after collection using the QIAamp mini kit (Qiagen, Hilden, Germany), following the manufacturer’s instructions. Serum samples were obtained and kept at − 80 °C until analysis.

### C. Trachomatis genotyping

DNA was extracted with the Cell Lysis Kit (Hybribio Corp, Guangdong, China) using negative and positive quality control products. A 200 bp conserved cryptic plasmid fragment of *C. trachomatis* DNA was amplified for diagnosis of *C. trachomatis* infection with the following primes: CT1: 5’-TTCCCCTTGTAATTCGTTGC-3’ and CT2: 5’-TAGTAACTGCCACTTCATCA-3’ as previously described [14]. *C. trachomatis* DNA positive samples were obtained for genotyping by Nested PCR [18]. An approximately 1,100-bp fragment encompassing *C. trachomatis omp1* gene was amplified using the outer primer sets for *omp1* P1: 5’-CTCAACTGTAACTGCGTATTT-3’ and omp1 P2: 5’- ATGAAAAAACTCTTGAAATCG-3’. A 580 bp VS1–VS2 fragment was further amplified using the inner primer sets P3: 5’- TGAACCAAGCCTTATGATCGACGGA-3’ and omp1 P2: 5’-TCTTCGAYTTTAGGTTTAGATTGA-3’ [14, 17]. Genotyping of the final PCR product (*omp1* VS1–VS2) was performed in Ruibo, Beijing (Supplementary Fig. S1).

### Mutation site analysis

The *omp1* VS1–VS2 sequence of clinical samples was compared using BLAST at the National Center for Biotechnology Information (http://www.ncbi.nlm.nih.gov). DNA Star software was used to depict the relationships between clinical isolates and the following reference strains of *C. trachomatis* obtained from GenBank (accession numbers in parentheses): B/B-16(AY950630), B/IU-1226 (AF063208), D/B120 (X62918), Da/TW-448(X62921), E/Bour(X52557), F/IC-Cal3(X52080), G/UW57/Cx (AF063199), H/Wash(X16007), J/UW36/Cx (AF063202), and K/UW31/Cx (AF063204) [2, 6].

### *C. Trachomatis* pgp3 antibody assay

Luciferase immunosorbent assay (LISA) was used to detect *C. trachomatis*-specific antigen pgp3, as described previously [19, 20], which was more sensitive than enzyme-linked immunosorbent assay (ELISA) [20]. Briefly, the *C. trachomatis* pgp3 gene was amplified and sub-cloned into the pNLF1-N luciferase expression vector (Promega, Madison, WI, United States) downstream of the Nluc luciferase gene, which was then transfected into HeLa cells. The cell lysates containing the Nluc-pgp3 fusion protein expressed in HeLa cells were harvested and confirmed using the anti-luciferase antibody. The 96-well white microplate were coated with 50 µL/well Protein G (5 mg/ml; Genscript, Nanjing, China) and incubated overnight at 4 °C. After washing and blocking, 50 µL diluted sera (1:100 dilutions in 2% non-fat dry milk) was added to the wells and incubated for 1 h at 37 °C, followed by washing five times. 50 µL Nluc-Pgp lysates were then added as detection antibody to each well and incubated at 37 °C for 30 min. After washing, 50 µL of the Nano-Glo Luciferase assay reagent was added to determine the luciferase light units of the Nano-Glo Luciferase assay by Fluoroskan Microplate Fluorometer (Thermofisher, United States). The cut-off value of anti-Pgp3 IgG LISA was derived from the average value of negative controls plus 3 standard deviations as described previously.

### Gynecological examination

The data obtained from gynecological examination, including routine leucorrhea, colposcopy, and cervical cytology, were collected to investigate the association between *C. trachomatis* infection, pgp3 antibody, *omp1* VS1–VS2 gene mutation and clinical manifestations.

The vagina cleanliness grade has been widely used in for gynecological studies [21] and can be used to evaluate the condition of the vaginal microenvironment. Routine leucorrhea was evaluated as described previously [22]. The vaginal swab was placed and mixed vigorously with 1 mL of sterile phosphate-buffered saline (PBS) in a tube, dripping one to two drops of the vaginal discharge specimen on slides, followed by immediate examination under an optical microscope for observation of white blood cells, red blood cells, *Trichomonas vaginalis*, epithelial cells, clue cells, and fungi. Vagina cleanliness was evaluated on the basis of bacterial morphology and microscopy. According to the vaginal bacteria, cocci, epithelial cell, and leukocytes, vaginal cleanliness was classified as grades I–IV. Classes I and II of vaginal cleanliness are considered normal, while classes III and IV are considered abnormal, with grade IV regarded as severe vaginal cleanliness.

Colposcopy was performed following the standard procedure [14]. According to the American Society for Colposcopy and Cervical Pathology (ASCCP), the colposcopy findings are characterized as benign, low-grade squamous intraepithelial lesion, high grade squamous intraepithelial lesion, and cancer.

Cervical cytology samples were obtained as described previously [23]. Cervical cytology was graded as Negative for Intraepithelial Lesion and Malignancy, atypical squamous cells (ASCIIS), low grade squamous lesion (L-SIL), high grade squamous lesion (H-SIL) and squamous cell carcinoma or adenocarcinoma (SCC/AC), corresponding to cervical pathology categories of normal, cervicitis, CIN grades I and II, CIN grade III, and cervical cancer, respectively [24].

### Statistical analysis

Statistical analyses were conducted using SPSS software (version 19.0; IBM Corp., Armonk, NY, USA). P values were determined using Chi-square tests (Pearson Chi-square, continuity correction, and Fisher’s exact tests) for categorical variables. Bivariate logistic regression was used to assess the risk factors affecting the prevalence of *C. trachomatis* and the relationship between recent or previous infection, *C. trachomatis omp1* VS1–VS2 gene sense mutations, and gynecological manifestations. Statistical differences were considered significant at *p* < 0.05.

## Results

### Prevalence of *C. Trachomatis* infection

**Fig. 1:**
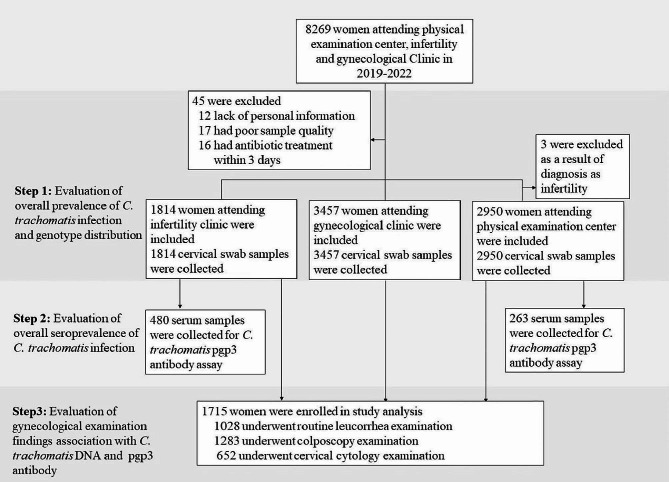
Study flow diagram

Between 2019 and 2022, 8,269 women were enrolled in our study; 48 participants were excluded due to poor sample quality, antibiotic treatment within 3 days, or missing personal information. Therefore, 8,221 women with available cervical swab samples were included in the final analysis (Figs. 1), 5006 of which were obtained from our previous study [14].

The overall prevalence of female reproductive tract *C. trachomatis* was 3.76% (309/8221). The highest prevalence of *C. trachomatis* infection was observed in women aged ≤ 20 years (12.87%; 13/101), which decreased with increasing ages up to 50 years (1.53%; 18/1176). Notably, the middle-aged women (aged 41–50 years) with infertility had an approximately two–fold higher prevalence of *C. trachomatis* infection than those presenting to the PEC (3.95% vs. 2.02%, *p* < 0.001), while women with infertility and those presenting to the PEC had similar prevalence rates in age groups of 31–40, 21–30, and < 20 years (Table 1). Bivariate logistic regression analysis showed that age is a risk factor for *C. trachomatis* infection (odds ratio [OR] = 9.496, 95% confidence interval [CI] = 4.505–20.013, *p* < 0.001) (Supplementary Table S1). These results suggest that young women may be more prone to *C. trachomatis* infection.

**Table 1:** Prevalence of *C. trachomatis* infection in different populations by age

Age(years)	Total [n (%)]	Groups [Posi n (%)]			χ^2^	P
		Infertility [Posi n (%)]	PEC [Posi n (%)]	Gynecological [Posi n (%)]		
≤ 20	13(12.87%)	2(14.29%)	1(16.67%)	10(12.3%)	11.231	0.004
21–30	133(5.65%)	26(4.46%)	21(4.45%)	86(6.55%)	59.023	< 0.001
31–40	91(3.44%)	24(2.47%)	29(3.25%)	38(4.88%)	3.319	0.190
41–50	54(2.76%)	9(3.95%)	18(2.02%)	27(3.24%)	9.002	0.011
> 50	18(1.53%)	1(2.94%)	13(1.89%)	4(0.88%)	12.996	0.002
Total	309(3.76%)	62(3.42%)	82(2.78%)	165(4.77%)	57.922	< 0.001
*χ* ^*2*^	166.388	45.903	26.293	128.485		
*P*	< 0.001	< 0.001	< 0.001	< 0.001		

PEC: physical examination center

### Distribution of *C. trachomatis* genotypes and *omp1* VS1-VS2 mutations

Sequencing of the *omp1* gene from amplified DNA of the 309 clinical strains revealed that *C. trachomatis* serovar E (85, 27.51%) was the most prevalent genotype, followed by F (72, 23.30%), J (60, 19.42%), D (45, 14.56%), G (22, 7.12%), H (11, 3.56%), K (11, 3.56%), Da (1, 0.32%), and B (2, 0.65%) (Table 2). 26.86% (83/309) of all clinical strains had genetic changes within *C. trachomatis omp1* gene VS1–VS2 segment, which occurred most frequently among genotypes J (98.3%, 59/60), K (45.45%, 5/11), H (27.27%, 3/11), G (9.09%, 2/22), and E (8.24%, 7/85), whereas genotype B showed no mutation, compared to the reference strains (Table 2). Moreover, no significant differences were observed in the distribution of *C. trachomatis* genotypes or mutations among women with infertility and other participants (data not shown). All mutations were double-checked using nested PCR and bi-directional DNA sequencing.

A detailed multiple sequence alignment of the sequences from the 309 clinical isolates was carried out to determine the variable nucleotide positions, which may cause amino acid replacements and potentially alter the function and antigenicity of *C. trachomatis omp1*. Among the 60 genotype J isolates, most (53/60, 88.33%) differed from the reference strain J/UW36 at the 369 position, similar to the sequence of the *C. trachomatis* J/isolate 6858 (AY950622) from China identified on BLAST searching. For genotypes D–H and K, most *omp1* gene sequences were identical to the corresponding sequences from reference isolates. One genotype D strain displayed C→T and G→A substitutions at positions 292 and 626, respectively. Four genotype K strains displayed an A→G substitution at position 293, and one strain demonstrated insertion of T at position 161 compared to K(UW31). Similarly, genotype Da sequences displayed a silent A→T substitution at position 369/636 compared to Da(TW-448). For genotype E, seven isolates differed from the reference strain E/Bour, among which the nucleotide substitutions occurred at positions 236, 286, 336, 377, 534, and 537 (changes in base pairs A to G, A to G, T to N, C to T, A to G, and T to N, respectively), resulting in amino acid changes from Asn to Ser, Thr to Ala, His to Gln, Ala to Val, Pro to Pro (silent), and Asp to Glu, respectively (Table 2).

To further investigate the evolutionary relationships among clinical isolates, a phylogenetic tree was constructed. Each *C. trachomatis* genotype formed a single clade. F and G were closely clustered and separated from the branch gathered by D and E (Supplementary Fig. S2).

**Table 2:** Distribution of *C. trachomatis* genotype and *omp1* VS1-VS2 mutations

Genotype	Prevalence [n (%)]	No. of mutations	Mutation		Amino acid change
			Nucleotide	Position	
D	45(0.54%)	1	C→T	292	H→Y
			G→A	626	G→E
		2	M	M	M
Da	1(0.01%)	1	A→T	636	Silent
E	85(1%)	1	A→G	236	N→S
		1	A→G	286	T→A
		1	T→N	336	H→Q
		1	C→T	377	A→V
		1	A→G	534	Silent
		1	T→N	537	D→E
		1	M	M	M
F	72(0.87%)	1	+C	204	+S
			+C	208	+L
		2	M	M	M
G	22(0.27%)	1	G→A	487	G→S
		1	M	M	M
H	11(0.13%)	1	A→G	272	N→S
			T→A	507	D→E
		2	M	M	M
J	60(0.73%)	53	C→T	369	Silent
		1	+C	150	Silent
			G→A	211	D→N
		1	C→T	302	T→I
		1	G→A	506	S→N
		3	M	M	M
K	11(0.13%)	4	A→G	293	D→S
		1	+T	161	+L
B	2(0.02%)	0	-	-	-
Total	309(3.76%)	83	-	-	-

M: More than three mutation sites. Amino acid change: H: Histidine (HIS); Y: Tyrosine (TYR); G: Glycine (GLY); E: Glutamic (GLU); N: Asparagine (ASN); S: Serine (SER); T: Threonine (THR); A: Alanine (ALA); Q: Glutamine (GLN); V: Valine (VAL); D: Aspartic (ASP); +S: add a Serine (SER); +L: add a Leucine (LEU); I: Isoleucine (ILE). -: Not available

### Seroepidemiological characteristic of *C. Trachomatis*

To determine the seroprevalence of *C. trachomatis*, serum samples were obtained from 743 women, including 480 women with infertility and 263 women who attended the PEC. The overall seroprevalence of *C. trachomatis* was 47.51% (353/743). The highest seroprevalence of *C. trachomatis* was 82% among women with infertility aged 31–40 years and 45.83% among PEC women aged 21–30 years (Table 3). There were no statistically significant differences in *C. trachomatis* seroprevalence among women with different *C. trachomatis* genotypes and *omp1* VS1-VS2 mutations (Supplementary Table S2).

**Fig. 2:**
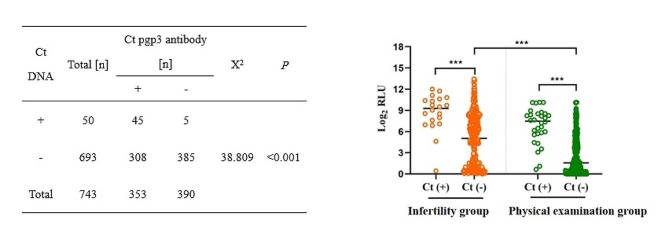
Distribution of *C. trachomatis p*gp3 antibody (log2 RLU) value among women with infertility and those presenting for routine physical examination. Serum samples were collected from 480 women with infertility and 263 women undergoing routine physical examination. There were no significant differences in the prevalence of *C. trachomatis* pgp3 between women with positive *C. trachomatis* DNA in the infertility (86.67%, 26/30) and routine physical examination (95%, 19/20) groups (*p* > 0.05). However, compared to women with positive *C. trachomatis* DNA, those with negative *C. trachomatis* DNA had a markedly higher *C. trachomatis* seroprevalence (56.74%, 261/460 vs. 20.17%, 47/233) (*p* < 0.001). Ct: *C trachomatis*; RLU: relative light unit

The *C. trachomatis* pgp3 antibody was detected more commonly in women with infertility (73.68%) compared to women attending to the PEC (27.76%) (Table 3), which was further confirmed by a case-control study among matched age and population groups (Supplementary Table S3). When the analysis was restricted to women with undetectable *C. trachomatis* DNA, the *C. trachomatis* seroprevalence was markedly higher among women with infertility compared to those presenting to the PEC (56.74% vs. 20.17%, *p* < 0.001) (Fig. 2; Table 3). However, both groups had similar prevalence rates of *C. trachomatis* DNA. An age-matched case-control study was performed to exclude the effect of age, which showed similar results (Supplementary Table S3). These data suggest that most women with infertility had current or past infection with *C. trachomatis*, indicating that recurrent infections occur commonly. Repeated infections with *C. trachomatis* can cause salpingitis, tubal occlusion, and subsequently secondary infertility. Thus, women with secondary infertility should be screened with *C. trachomatis* DNA and antibody screening.

**Table 3:** Seroprevalence of *C. trachomatis* pgp3 antibody among physical examination and women with infertility by age

Classification	Total [ Posi n (%)]	Prevalence [n (%)]		χ^2^	P
		Infertility [ Posi n (%)]	PEC [ Posi n (%)]		
Total [n (%)]	353(47.2%)	280 (73.68%)	73 (27.76%)		< 0.001
≤ 20	2(50%)	2 (66.67%)	0 (0%)	66.524	< 0.001
21–30	88(61.12%)	77 (64.17%)	11 (45.83%)		
31–40	191(50.13%)	164 (82%)	27 (33.33%)		
41–50	60(35.91%)	37 (64.19%)	23 (20.91%)		
> 50	12(25.55%)	-	12 (25.53%)		
*X* ^*2*^	326.504	330.679	31.316		
*P*	< 0.001	< 0.001	0.015		
Ct DNA negative population	310(44.7%)	261(56.74%)	49(20.59%)		< 0.001
≤ 20	2(0.65%)	2(66.67%)	-	72.772	< 0.001
21–30	75(24.19%)	68(61.82%)	7(33.33%)		
31–40	170(54.83%)	155(53.26%)	15(22.73%)		
41–50	53(17.10%)	36(64.29%)	17(16.35%)		
> 50	10(3.23%)	-	10(21.74%)		
*χ* ^*2*^	293.839	312.736	18.653		
*P*	< 0.001	< 0.001	< 0.001		

Ct: *C. trachomatis*; PEC: physical examination center; -: Not available

Women with *C. trachomatis* DNA were more likely to have cervical abnormalities compared to those with undetectable *C. trachomatis* DNA (54.70% vs. 22.07%, *p* < 0.001) (Table 4). Moreover, in the multivariate model, leucorrhea cleanliness (OR = 3.653, 95% CI = 2.662–5.011, *p* < 0.001), CIN on cervical cytology (OR = 4.087, 95% CI = 2.284–7.315, *p* < 0.001), and CIN on colposcopy (OR = 3.815, 95% CI = 1.768–8.235, *p* < 0.001) were independently associated with detectable *C. trachomatis* DNA. Stratification analysis further confirmed that women who tested positive for both *C. trachomatis* DNA and antibody had increased risks of cervical abnormalities (OR = 1.747, 95% CI = 1.096–2.783, *p* = 0.019). However, testing positive only for *C. trachomatis* pgp3 antibody was not significantly associated with cervical abnormalities (Table 5 and Supplementary Table S4).

Although a higher rate of cervical abnormalities was found among women with *C. trachomatis omp1* mutations, the results were not statistically significant. Notably, sense mutations were associated with CIN on colposcopy (OR = 6.033, 95% CI = 1.219–39.185, *p* = 0.045) (Supplementary Table S5 and S6).

**Table 4:** Gynecological examination findings of women with respect to *C. trachomatis* DNA and *C. trachomatis* pgp3 antibody

Characteristic	Ct DNA [n]		Total [n]	χ^2^	P	Ct pgp3 [n]		Total [n]	χ^2^	P
	+	-				+	-			
Cervical abnormality^a^										
+	157	316	473	128.216	< 0.001	87	57	144	1.003	0.986
-	129	1113	1429			216	142	258		
Leucorrhea cleanliness										
+	139	274	413	69.037	< 0.001	82	51	133	0.006	0.939
-	75	540	615			147	93	240		
Cervical cytology CIN										
+	18	11	29	13.207	< 0.001	3	3	6	0.009	0.919
-	187	436	623			213	139	358		
Colposcopy CIN										
+	18	44	62	25.556	< 0.001	5	7	12	1.091	0.296
-	112	1109	1221			274	176	450		

Ct: *C. trachomatis*; CIN: cervical intraepithelial neoplasia; ^a^ Cervical abnormality: any abnormality in leucorrhea cleanliness, cervical cytology CIN, or colposcopy CIN.

**Table 5:** Association of *C. trachomatis* DNA and *C. trachomatis* pgp3 antibody with gynecological examination findings

Symptoms	Ct DNA +		P	Ct pgp3 antibody +		P
	NO.	OR (95%CI)		NO.	OR (95%CI)	
Leucorrhea cleanliness	1028	3.653 (2.662–5.011)	< 0.001	373	1.009 (0.786–1.296)	0.943
Cervical cytology CIN	652	4.087 (2.284–7.315)	< 0.001	358	0.443 (0.095–1.982)	0.281
Colposcopy CIN	1283	3.815 (1.768–8.235)	< 0.001	462	0.585 (0.087–3.941)	0.582

Ct: *C. trachomatis*; CIN: cervical intraepithelial neoplasia; OR: odds ratio

## Discussion

The prevalence rates of *C. trachomatis* DNA and pgp3 antibody in a representative sample of Southern Chinese women aged 13–87 years were 3.76% and 47.46%, with the highest DNA prevalence among women aged 14–20 years and the highest seroprevalence rate among women aged 21–30 years, which was in line with the results reported from developing countries [7]. A relatively low pgp3 seropositive rate of 28.1% was previously reported among individuals aged 18–65 years from northern China [19], which may be due to an unbalanced age distribution and regional differences.

In our analysis, age was closely associated with *C. trachomatis* infection. We found a high prevalence of *C. trachomatis* infection in young women aged < 30 years, with the prevalence decreasing at older ages. The highest prevalence was reported in adult women aged 18–24 years in the general population in Germany [19]. In southwest China, *C. trachomatis* infection was more common in women aged 25–34 years [23], suggesting that young women were more susceptible to *C. trachomatis* infection, which may be due to their sexual activities, low educational status, absence of appropriate sexual education, and poor vaginal hygiene [25]. Tailored counseling coupled with annual screening may decrease the burden of *C. trachomatis* infection among the young population.

In this study, we found that *C. trachomatis* serovar E was the most prevalent *C. trachomatis* genotype in the female lower genital tract, which was congruent with results reported from Sweden, Stockholm County, and Finland [3, 18], while serovar D was the most common in Liuzhou and serovar F in Thailand [1]. Furthermore, we found that F was the most prevalent (24%) in women with positive *C. trachomatis* pgp3 antibody. Genotype F was associated with a higher urogenital infections loads and greater disease progression [26]. A high titer of antibody against the powerful *C. trachomatis* pgp3 antigen may be secreted during infection, which can lead to an autoimmune response.

*C. trachomatis omp1* is evolutionarily highly conserved and is thought to play a vital role in protective immunity, which consists of 5 regions of conserved sequence that alternate with 4 variable regions (VS1–VS4). The *omp1* VS1-VS2 are surface exposed and allow genotype classifications, which may alter its function, antigenicity and clinical manifestations [27, 28]. We found 83 genetic variants of the 309 *omp1* gene sequences, in accordance with previously reported genetic variability of 10–81% [29]. Genotype J was one of the most mutable genotypes with a mutation rate of 98.3%. The identification of mutations at positions 150 and 506 in genotype J is a new finding. Although the mutations occurred at the position 369 diverging from the commonly used reference strain J/UW36, they were confirmed to be identical to the C. trachomatis J/isolate 6858 and Taiwanese genotype J strains [2]. For the most prevalent genotype E, seven samples diverged (7/85, 8%) from the reference sequence E/Bour ( the most constant genotype), which is in line with a prior study reporting the genetic variants of E strains [3].

With the exception of genotype B, mutations also occurred among other *C. trachomatis* genotypes, with 33% (28/83) of them being sense mutations, leading to an elongated protein. Surprisingly, no statistical differences in clinical characteristics were observed between mutation and non-mutation groups, while sense mutations were more likely to result in greater severity of CIN on colposcopy. Several studies have reported the incidence of *C. trachomatis* infection over the last two decades. In our phylogenetic analysis, *omp1* polymorphism was relatively stable, and the rate of genetic change was slow [5].

Our study is unique because we assessed *C. trachomatis* DNA and antibody concurrently from clinical samples, which is vital for epidemiological and vaccination studies. Pgp3-based LISA used in this study was validated to be a suitable assay for the detection of anti-*C. trachomatis* antibody as our previously reported [28]. As anticipated, women with positive *C. trachomatis* DNA had a markedly higher prevalence of pgp3 antibody compared to those with negative *C. trachomatis* DNA. We observed an association between cervical abnormalities and *C. trachomatis* DNA, but not with the pgp3 antibody. Therefore, noninvasive nucleic acid amplification tests are recommended to detect *C. trachomatis* infection of the genital tract [30]. Antibody responses can indicate the presence of chlamydial antigens in the host, indicating past infections. Thus, seropositive women often have no obvious clinical manifestations, which may explain the lack of significant associations between the pgp3 antibody and cervical abnormalities. Intriguingly, the pgp3 antibody persists in the human body for as long as 12 years [31], which can be used to estimate the cumulative risk of *C. trachomatis* infection; considering this, our results suggest that almost half of the women in our cohort had been infected with *C. trachomatis*.

*C. trachomatis* infection often causes few or mild symptoms, making them undetectable. Worsening and persisting infections may result in scarring and obstruction of the fallopian tubes, ultimately leading to female infertility. We found that women with infertility had a similar prevalence of *C. trachomatis* DNA with those of childbearing age attending the PEC. However, a markedly higher seroprevalence was observed in in women with infertility, which was consistent with previous studies that women with infertility have 2–3 times higher levels of *C. trachomatis* antibodies compared to the general female population [32]. These findings suggest that the *C. trachomatis* antibody test is a useful predictor of *C. trachomatis*-mediated infertility, as most women with infertility had previous *C. trachomatis* infection. Recurrent infections may also increase the risk of *C. trachomatis* spreading from the genital tract to the gastrointestinal tract, leading to long-lasting colonization, and intensify the chlamydial pathogenicity in the reproductive system when it is transmitted back to the genital tract, similar to a second hit [33]. These results highlight the usefulness and significance of *C. trachomatis* screening, including genital and intestinal *C. trachomatis* nucleic acid and antibody toward *C. trachomatis*, for infertility.

There were several limitations to our study. First, a large female population was randomly recruited from a single hospital, which could limit the generalizability of our findings. Second, there was a lack of detailed information on demographic characteristics, such as such as ethnicity, educational level, country of birth, and sexual activities, which may have introduced bias in the prevalence estimates. Third, data were collected for only 2.5 years, whereas the distribution of *C. trachomatis* genotypes may have been different if additional data were collected from a larger sample and over a longer time period. Fourth, we did not collect information related to the gynecological examination and pgp3 antibodies for all participants because most refused to undergo the test, which may have impacted our results related to the association between *C. trachomatis* infection and cervical abnormalities.

In conclusion, we conducted the first investigation into the point DNA prevalence and seroprevalence of *C. trachomatis* among women in China, providing estimates for both current infection and cumulative exposure. Overall, the *C. trachomatis* prevalence was high. The difference between seroprevalence (47.46%) and current DNA prevalence (3.76%) in our study reflects the high clearance of *C. trachomatis* infections. The seroprevalence among women with infertility with negative *C. trachomatis* DNA was more than two-fold higher compared to that in the PEC group. These findings are helpful in elucidating the association between *C. trachomatis* and infertility. We also demonstrated a strong correlation between positive *C. trachomatis* DNA and leucorrhea cleanliness, CIN on cervical cytology, and CIN on colposcopy. These data bear important implications for the continuous improvement of primary and secondary prevention strategies as well as the development of subunit vaccines to decrease *C. trachomatis* infections in China.

### Electronic supplementary material

Below is the link to the electronic supplementary material.

Supplementary Material 1

## Data Availability

The datasets used and/or analyzed during the current study are available from the corresponding author on reasonable request.

## Abbreviations

CIN: cervical intraepithelial neoplasia
VS (1: 4)-variable segments (1-4)
PEC: physical examination center
ASCCP: American Society for colposcopy and cervical pathology
LSIL: low-grade squamous intraepithelial lesion
HSIL: high grade squamous intraepithelial lesion
NTLM: Negative for Intraepithelial Lesion and Malignancy
ASCIIS: atypical squamous cells
L: SIL-low grade squamous lesion
H: SIL-high grade squamous lesion
SCC/AC: squamous cell carcinoma or adenocarcinoma
CI: confidence interval
