# Supplementary material for: Characterization of genital chlamydia trachomatis infection among women attending infertility and gynecology clinics in Hunan, China
Source: BMC Infect Dis. 2024 Apr 15;24:405. doi: 10.1186/s12879-024-09254-8 (PMC11020174; doi:10.1186/s12879-024-09254-8)
Supplement: Supplementary file 1 — Supplementary Material 1 [file 12879_2024_9254_MOESM1_ESM.docx]

**Supplementary**


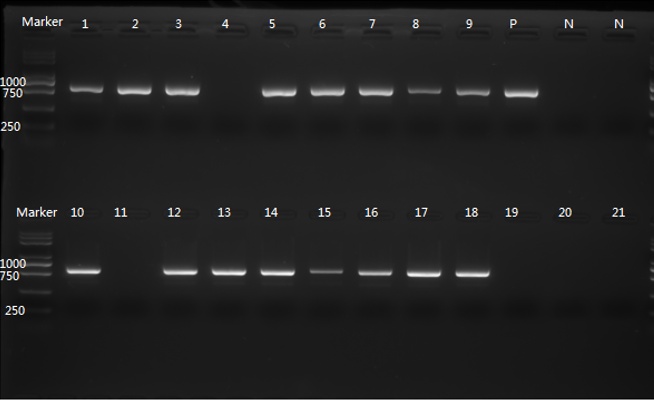


**Figure S1.** **PCR amplification products of *C. trachomatis* *omp1* VS1-VS2 from clinical samples.** N: negative control(ddH_2_O); P: positive control; 1-3、5-10、12-18: *C. trachomatis* *omp1* VS1-VS2 positive samples; 4、11、19-21: *C. trachomatis* *omp1* VS1-VS2 negative samples.

**Figure S2. Evolutionary tree of *C. trachomatis* *omp1* VS1-VS2 fragments from 309 clinical strains and 9 standard strains.** Genotype F was closely related to genotype G, the genotype H was closely related to genotype K and J, but not to those from between F and E.

**Table S1.** **Associations of age with *C. trachomatis* infection by** **multivariate analysis.**

| Age(years) | *C. trachomatis* DNA positive | *P* |
| --- | --- | --- |
|  | OR (95%CI) |  |
| <20 | 9.496(4.505-20.013) | <0.001 |
| 21-30 | 3.849(2.341-6.329) | <0.001 |
| 31-40 | 2.295(1.377-3.823) | <0.001 |
| 41-50 | 1.830(1.068-3.135) | 0.028 |
| >50 | 1(Reference) | <0.001 |

OR: odds ratio; CI: confidence Interval.

**Table S2**. **Genotype distribution and *omp1* VS1-VS2 mutation of *C. trachomatis* stains by *C. trachomatis* pgp3 antibody**

Ct: *C. trachomatis*; -：Not available.

| Classification | Ct pgp3 antibody [n (%)] | | | Total [n ] | | *X^2^* | *P* |
| --- | --- | --- | --- | --- | --- | --- | --- |
|  | + | - | |  |  |  |  |
| **Genotype** | | | | | | 2.976 | 0.809 |
| E | 8 | 2(4%) | 10 | | |  |  |
| F | 12 | 1(2%) | 13 | | |  |  |
| J | 10 | 1 | 11 | | |  |  |
| D | 6 | - | 6 | | |  |  |
| G | 4 | 1 | 5 | | |  |  |
| H | 2 | - | 2 | | |  |  |
| K | 2 | - | 2 | | |  |  |
| B | - | - | 0 | | |  |  |
| Da | 1 | - | 1 | | |  |  |
| Total | 45 | 5 | 50 | | |  |  |
| **Omp1 VS1-VS2** | | | | | | | |
| Mutation | 14 | 1 | 15 | | 0.265 | | 0.607 |
| Non-mutation | 31 | 4 | 35 | |  |  |  |
| Total | 45 | 5 | 50 | |  |  |  |

**Table S3. Age-Matched Case-control study on the prevalence of *C. trachomatis* among physical examination and infertility participant.**

| Groups | Sample No. [n] | Age(years) | Ct DNA  [Posi n (%)] | Ct pgp3 antibody  [Posi n (%)] |
| --- | --- | --- | --- | --- |
| Infertility population | 153 | 36.41±5.634 | 5(3.27%) | 98(64.05%) |
| PEC examination population | 153 | 36.41±5.496 | 24 (15.69%) | 44(28.76%) |
| *X^2^* |  |  | 12.448 | 20.535 |
| *P* |  | 0.798 | <0.001 | <0.001 |

Ct: *C. trachomatis;* PEC: physical examination center.

**Table S4. Relationship of *C. trachomatis* pgp3 antibody and *C. trachomatis* DNA detection with gynecological examination findings of female**

| Symptoms | Ct *+＆* pgp3+ | *P* | Ct *-＆* pgp3 + | *P* | Ct *+＆* pgp3 - | *P* | Ct *-＆* pgp3 - | *P* |
| --- | --- | --- | --- | --- | --- | --- | --- | --- |
|  | OR (95%CI) |  | OR (95%CI) |  | OR (95%CI) |  | OR (95%CI) |  |
| Leucorrhea cleanliness + | 1.747(1.096-2.783) | 0.019 | 0.779(0.624-0.974) | 0.029 | 2.883(0.564-14.739) | 0.204 | 0.875(0.693-1.105) | 0.263 |
| Cervical cytology CIN + | 0(0) | 0.999 | 0.555(0.108-2.859) | 0.482 | 0(0) | 0.999 | 1.143(0.275-4.759) | 0.854 |
| Colposcope CIN + | 2.355(0476-11.660) | 0.294 | 0.110(0.014-0.815) | 0.034 | 0(0) | 0.999 | 1.172(0.384-3.576) | 0.781 |

Ct: *C. trachomatis*; CIN: cervical intraepithelial neoplasia; OR: odds ratio; CI: confidence Interval.

**Table S5.** **Gynecological examination findings of female with *C. trachomatis* omp1 VS1-VS2 mutation and non-mutation**

| Symptoms | Total[n] | Mutation[n] | Non-mutation[n] | X^2^ | *Ρ* |
| --- | --- | --- | --- | --- | --- |
| Colposcopy CIN |  |  | | | 0.749 |
| + | 18 | 6 | 12 | 0.151 | 0.697 |
| - | 111 | 32 | 79 |  |  |
| Cervical cytology CIN |  |  | | | |
| + | 18 | 8 | 10 | 2.770 | 0.096 |
| - | 187 | 44 | 143 |  |  |

CIN: cervical intraepithelial neoplasia.

**Table S6. Gynecological examination findings of female with *C. trachomatis* *omp1* VS1-VS2 sense and nonsense mutations**

| Symptoms | Total [n] | Sense mutations  [n] | Nonsense mutations  [n] | X^2^ | *P* | Sense mutations | *Ρ* |
| --- | --- | --- | --- | --- | --- | --- | --- |
|  |  |  |  |  |  | OR (95%CI) |  |
| Colposcope CIN |  |  | | | | | |
| + | 6 | 4 | 2 | 4.060 | 0.043 | 6.033  (1.219-39.185) | 0.045 |
| - | 33 | 8 | 24 |  |  | 1 (reference) |  |
| Cervical cytology  CIN |  |  | | | | | |
| + | 8 | 4 | 4 | 0.212 | 0.645 | 1.933  (0.423-8.836) | 0.395 |
| - | 44 | 15 | 29 |  |  | 1 (reference) |  |

CIN: cervical intraepithelial neoplasia; OR: odds ratio; CI: confidence Interval.
